# Internal Jugular Vein Avulsion Controlled With Intraoperative Foley Balloon Occlusion Following Blunt Neck Trauma

**DOI:** 10.7759/cureus.70925

**Published:** 2024-10-06

**Authors:** Patrick D Melmer, Alexander Simmonds, Michel Aboutanos

**Affiliations:** 1 Acute Care Surgery, Virginia Commonwealth University, Richmond, USA; 2 General Surgery, Virginia Commonwealth University, Richmond, USA; 3 Surgery, Virginia Commonwealth University, Richmond, USA

**Keywords:** blunt trauma neck, foley's catheter, internal jugular ligation, neck trauma, tamponade balloon

## Abstract

Internal jugular vein injuries caused by blunt trauma are rare. Here, we describe the case of a severe internal jugular vein injury high in neck zone II and immediately lateral to the hypoglossal nerve, making surgical management extremely challenging. A Foley balloon inserted into the cranial end of the internal jugular vein allowed for effective hemorrhage control while the vein could be suture ligated. Balloon tamponade was helpful in a field with distorted and blood-stained anatomy. It allowed critical time to carefully dissect around the hypoglossal nerve and avoid the sequalae of its injury.

## Introduction

Internal jugular vein injuries caused by blunt trauma are rare [1]. Repair is preferred, if possible, although ligation is accepted in cases of uncontrollable hemorrhage [2-4]. Here, we describe the case of a severe internal jugular vein injury high in neck zone II and immediately lateral to the hypoglossal nerve, making surgical management extremely challenging. A Foley balloon inserted into the cranial end of the internal jugular vein injury allowed for effective hemorrhage control. In contrast, the vein could be suture ligated while decreasing the chance of iatrogenic injury to the hypoglossal nerve.

## Case presentation

**Figure 1:**
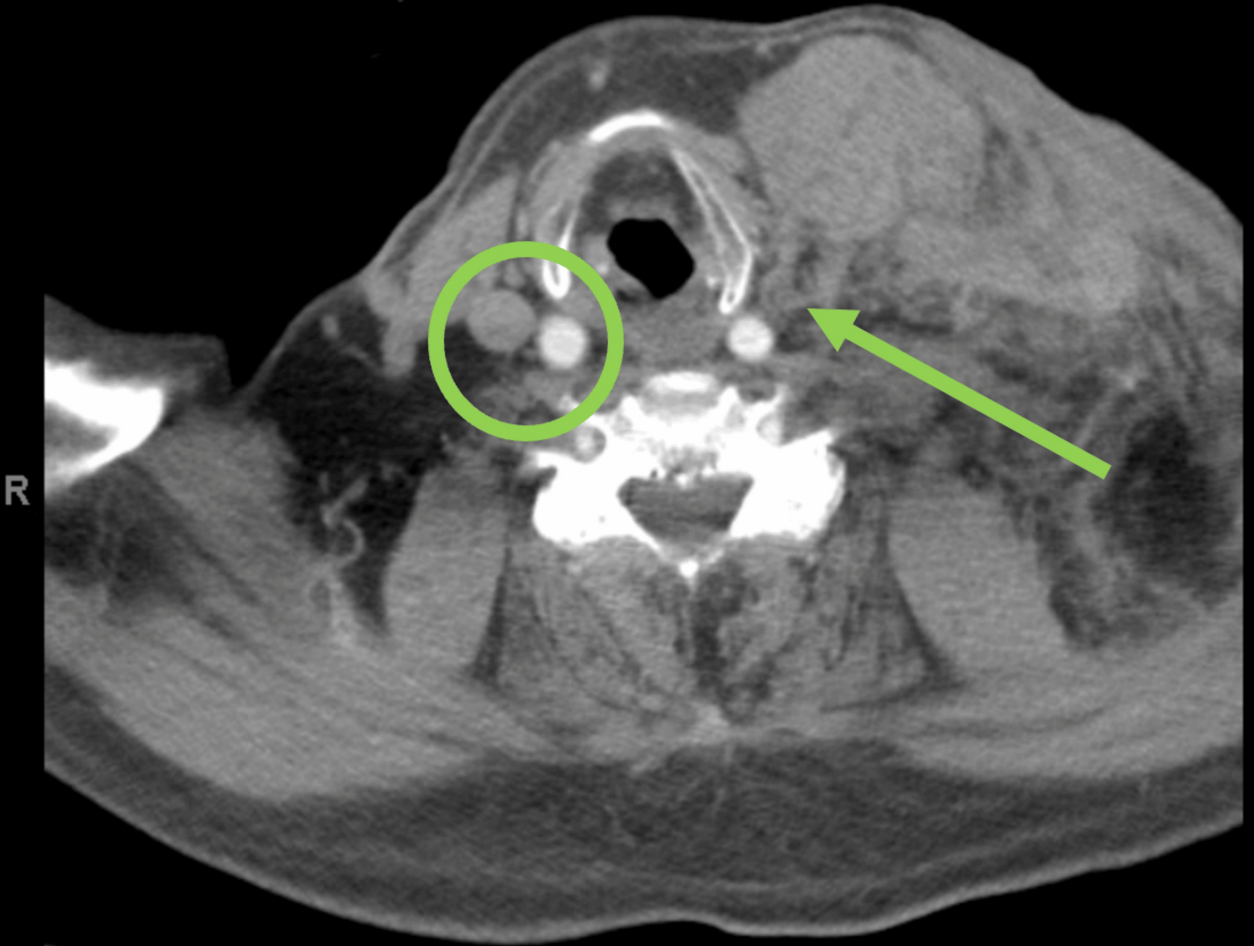
Neck computer tomography angiogram. Neck CTA demonstrating opacified bilateral carotid arteries, patent right internal jugular vein (green circle), and disrupted left internal jugular vein along with massive left neck hematoma (green arrow). CTA: computer tomography angiogram.

An 86-year-old male presented following a high-speed motor vehicle crash against a tree and was alerted as a high-level trauma for potential severe injuries. Per emergency medical responders, he had normal blood pressure throughout transport. On arrival, he had a very large left neck hematoma with no hard signs of vascular trauma (Figure 1). He reported taking apixaban and was administered prothrombin complex concentrate as part of his resuscitation. His primary survey demonstrated an intact airway and no evidence of hemodynamic instability, so he was deemed safe for computer tomography imaging. On imaging, he had active extravasation from the left internal jugular vein (Figure 1).

**Figure 2:**
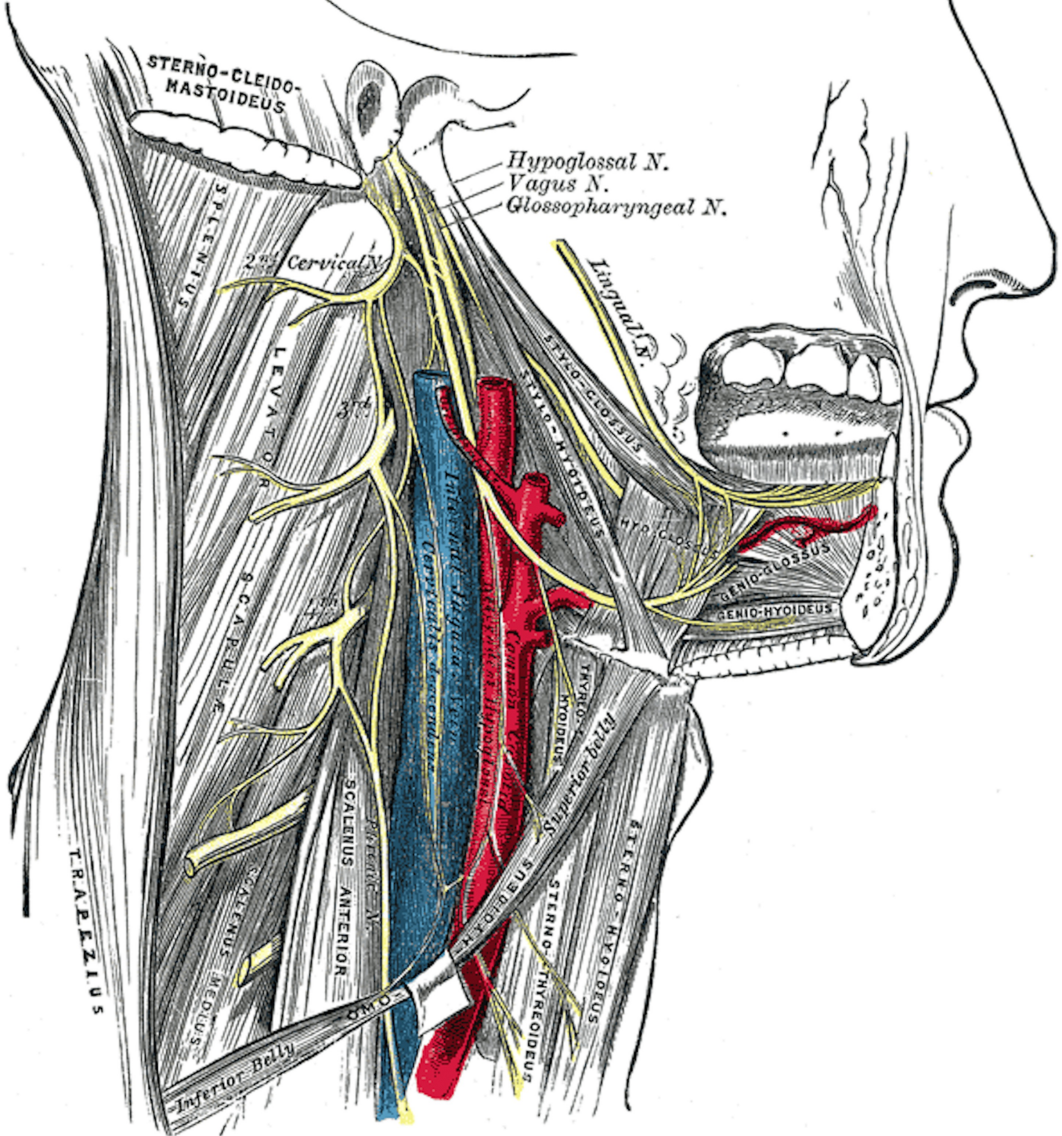
Anatomic illustration demonstrating the close relationship of the hypoglossal vein to the carotid artery and internal jugular vein. Hypoglossal nerve, cervical plexus, and their branches. Adapted from Henry [5].

With the concern for ongoing bleeding and the potential for mass effect on his airway, he was taken emergently to the operating room for neck exploration. A neck exploration along the anterior border of the sternocleidomastoid muscle was performed. Here, the left internal jugular vein was noted to be avulsed nearly in half, high, and immediately lateral to the hypoglossal nerve with a large amount of ongoing hemorrhage from the cranial side. With blood obscuring the field from this venous injury immediately adjacent to the hypoglossal nerve, there was a heightened concern that venous ligation would cause inadvertent injury to the nerve (Figure 2).

**Figure 3:**
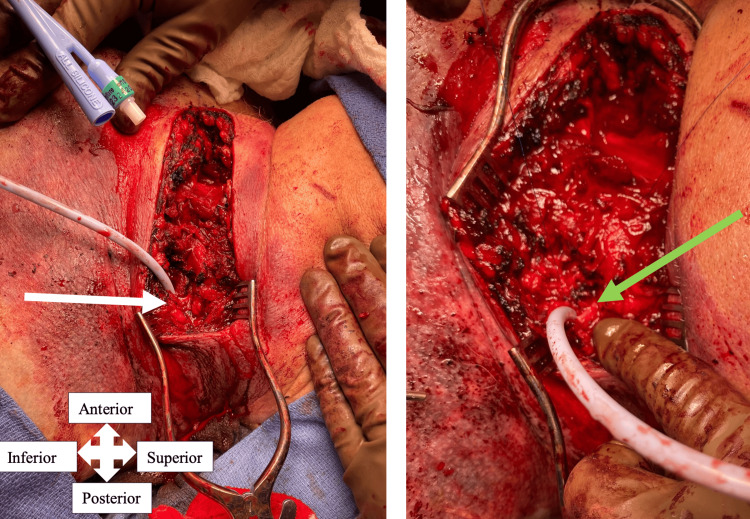
Intraoperative photos. Demonstrating foley placement into the cranial portion of the avulsed left internal jugular vein (white arrow) and close position to the hypoglossal nerve (green arrow).

A 14Fr Foley catheter (CR Bard, Inc., Murray Hill, NJ) was slowly advanced into the vessel lumen and inflated with saline, with excellent subsequent hemostasis (Figure 3). This allowed time and visualization for careful dissection around the nerve, which was done sharply with scissors to avoid thermal injury to the nerve. The back wall of the internal jugular vein was transected, leaving a cuff of healthy, non-injured vein that was sewn to its anterior portion, completing ligation of the cranial portion and obtaining hemostasis. With definitive hemorrhage control obtained, the foley was removed. The caudal end was noted to be largely thrombosed but was also suture ligated to decrease the chance of rebleeding. The carotid artery and its branches were uninjured and protected throughout the dissection. The neck was widely drained and closed in layers. He was taken to the intensive care unit for further resuscitation. His convalescence was uneventful, and he had no evidence of hypoglossal nerve injury. He was discharged home, and his drain was later removed in the clinic.

## Discussion

Blunt trauma leading to internal jugular vein avulsion is very rare [2]. Similar to arterial injuries in the neck, venous repair is preferred if possible. This may be accomplished by direct suture repair of an injury. However, ligation is an accepted approach for patients in extremis, with the key goal of controlling hemorrhage to save a patient's life. Other less common techniques, including endovascular stenting or compression of the region, have been reported as successful in isolated cases [1,4]. In our patient, his acute trauma and risk for airway compromise necessitated an open approach. A neck exploration demonstrated the injury immediately next to the hypoglossal nerve. Inadvertent manipulation of the nerve, let alone transection or ligation, could lead to devastating consequences such as tongue paralysis and permanent dysphagia.

Furthermore, visualization proved challenging due to the torrent of bleeding on anticoagulation. Balloon tamponade was helpful in a field with distorted and blood-stained anatomy. It allowed critical time to carefully dissect around the hypoglossal nerve and avoid the sequalae of its injury. This technique has been described in anatomic regions of nonjunctional hemorrhage, such as the axilla, where compression against a fixed point is less feasible. It has also been utilized in austere environments, such as military transport, where temporary occlusion allows critical time for transport to a higher level of operative care [6]. We highlight that foley balloons are readily available in most emergency, operative, or other healthcare facilities and are inexpensive. They do not require special stocking or ordering, and the chance of staff being unfamiliar with their use will be rare. Possible complications include those that may occur with any manipulation of vessels, such as rupture, dissection, or prolonged occlusion leading to ischemia. Familiarity with vascular and endovascular techniques will mitigate these risks. Care following placement can range from temporary usage and removal after definitive hemorrhage control, as was in our case, to the catheters being sutured in place to the skin and left in place for some time before interrogation and removal. In these cases, titratable control is easy, as the balloon can be let down and observed for continued hemorrhage, and if present then reinflated. 

## Conclusions

Temporary Foley tamponade can serve as a valuable hemorrhage control asset in a variety of settings, from the operating room to the battlefield. They are versatile and readily available tools that are already present in most medical facilities, but if one is not available, other similar balloon catheters can serve the same purpose. They are inexpensive, allow for titratable hemorrhage control that can be managed at the bedside, and can act as a bridge to definitive treatment in austere conditions. We recommend this modality and hope to add to the growing body of literature that supports its use.
